# Acting out when psychosocial safety climate is low: understanding why middle-level managers experience upward mistreatment

**DOI:** 10.3389/fpsyg.2024.1336130

**Published:** 2024-04-17

**Authors:** May Young Loh, Maureen Frances Dollard

**Affiliations:** Psychosocial Safety Climate Global Observatory, Centre for Workplace Excellence, University of South Australia, Adelaide, SA, Australia

**Keywords:** psychosocial safety climate, upward bullying, workplace mistreatment, conflict escalation, retaliation

## Abstract

**Introduction:**

Upward mistreatment, despite being under studied, is an influential phenomenon affecting middle managers’ well-being and performance. The work environment hypothesis of bullying proposes that an undesirable work context is the main cause of workplace bullying, suggesting the importance of creating an anti-mistreatment climate, that is, psychosocial safety climate (PSC). In this study, we argue that upward bullying and aggression are unsafe behaviors, a “retaliation” by employees resulting from their unsafe work context.

**Methods:**

Using a large-scale multisource sample collected from 123 organizations, 6,658 middle managers and 34,953 employees, we examined the relationship between collective PSC, individual-perceived PSC and middle managers’ experience of upward mistreatment.

**Results:**

Single-level and multi-level modeling results suggested that PSC is an important element in reducing the likelihood of upward bullying and aggression, in turn, protecting managers’ well-being. More importantly, upward bullying is a way that employees act out when there is an undesirable working context.

**Discussion:**

Future research on workplace mistreatment should examine PSC and upward mistreatment. Interventions provided should focus on improving PSC which could in turn preventing upward mistreatment, thereby improving psychosocial safety for both employees and middle managers to prevent negative actions.

## 1 Introduction

Workplace mistreatment, such as bullying and aggression, are detrimental to employees, with enormous consequences, such as employees’ negative well-being, poor performance, harmful working relationships and deteriorating health, as well as the creation of an unsafe work environment (Boudrias et al., 2021; Hogh et al., 2021). Prior studies have identified various forms of workplace mistreatment including aggression, bullying, harassment, mobbing (i.e., a form of group bullying), incivility, and abusive supervision (Einarsen et al., 2012). There is a debate on the definitions of these constructs and whether they are distinct from one another, for example, bullying is often defined as a negative act that persistent for a certain period, while incivility involves low intensity actions and behaviors toward others. The proliferation of these constructs both advances and complicates the study of negative social interaction at work (Hershcovis, 2011). Although these behaviors differ in their nature, due to their frequency, intensity, and ways of interaction, they are forms of “workplace mistreatment,” herewith we termed bullying and aggression to refer to these negative acts. Workplace mistreatment in general is quite prevalent with meta-analysis reporting an average 34% of employees have personally experienced one of these negative actions (Dhanani et al., 2021). While scholars mostly agree that bullying involves negative acts that persistent in a long term and widely occur at workplaces where there is a power imbalance between the victim and the perpetrators, aggression can involve episodes of verbal or non-verbal deviant behaviors and actions with harmful intention. Both bullying and aggression are commonly experienced by workers and required prompt actions.

While research has found that most victims are likely to be employees in positions at the lowest level, a few studies have demonstrated that workplace bullying and aggression could be directed to anyone in an organization, regardless of their job position. For example, Björklund et al. (2019) found that 10% of managers experienced bullying and 11.4% of workers admitted that they behaved aggressively toward their supervisor in a recent study (Lyubykh et al., 2022). In a similar vein, a study conducted in India found that more than 50% of managers experienced repetitive negative actions from their superior, co-workers or subordinates (Rai and Agarwal, 2017). Moreover, in examining a newly emerging type of bullying, Forssell (2016) reported that individuals in supervisory positions are more likely to be exposed to cyberbullying. Although under studied, upward mistreatment is an influential phenomenon affecting managers’ well-being and performance. Without research on managers’ experience of bullying and aggression, the understanding of the mistreatment phenomenon in organizations is incomplete (Hoel et al., 2001), particularly among the middle-level managers who possess responsibility to take care of their workers and fulfill organizational goals. Specifically, scholars have called for more research related to bullying experienced by managers from their subordinates, with this termed “upward bullying” (Branch et al., 2021). We adapted this term and studying upward mistreatment in the current study. Surprisingly, although attention on the upward mistreatment issues is increasing, little evidence on why it occurs and how to prevent the situation. In this study, we aim to better understand managers’ experience of upward mistreatment, as well as the antecedents and consequences of these behaviors.

In the current study we examine how organizational factor could impede workplace mistreatment. The *work environment hypothesis* of bullying proposes that an undesirable work context is the main cause of workplace mistreatment (Hauge et al., 2007; Skogstad et al., 2011). A recent study among 48,537 Finnish workers found that mistreatment was difficult to stop, once it had started (Ervasti et al., 2023), highlighting the importance of studying the antecedents and leading indicators of these negative behaviors. To build an anti-mistreatment work context, we propose psychosocial safety climate (PSC), an organizational climate that is a leading indicator for employees’ psychological health and work stress. Psychosocial safety climate (PSC) is defined as employees’ perception of organizational policies, practices, and procedures in relation to their psychological health and safety issues (Dollard and Bakker, 2010). A high level of PSC is accompanied with ample resources and manageable demands, as well as an efficient psychosocial risk management system (Dollard et al., 2017; Loh et al., 2020). PSC and the associated work environment hypothesis could serve in at least two ways: firstly, by preventing the occurrence of mistreatment through establishing a desirable work context with less task-level psychosocial risks; and, secondly, by quickly resolving conflict and avoiding its escalation into bullying or more intense negative interactions. Stated simply, PSC is a leading indicator and a preventive measure of workplace mistreatment.

The present study contributes to the literature in three ways. Firstly, we expand the understanding on upward mistreatment by assessing the antecedent of why employees engage in such a negative act toward their leaders. We argue that this is closely related to their experience toward workplace climate and is a form of retaliatory responses. Specially, we examine the relationship between an anti-mistreatment climate (i.e., PSC) and the upward bullying and aggression experienced by middle-level managers. Secondly, despite focusing solely on the victims, we include both the perceptions of employees and managers using two sources of response to test relationships. While multisource data mitigates the possibility of common method bias and provides reliable results, understanding the perceptions from employees will help to answer the underlying mechanism of upward mistreatment. Thirdly, we examine how upward mistreatment relates to middle managers’ well-being. Using multilevel multisource data, we aim to expand the literature on upward mistreatment by examining its potential antecedents and its consequences for middle managers.

### 1.1 Managers’ experience of workplace mistreatment

The importance of middle managers’ roles in organizations is widely recognized. Middle-level managers are expected to respond to many expectations from both the top and bottom of an organization. On the one hand, middle-level managers are required to prioritize organizational goals set by executives. On the other hand, they are expected to take care of their subordinates. Managers’ well-being is found to be closely associated with their managerial quality (Parent-Lamarche and Biron, 2022), their interaction with other members of the organization (Zumaeta, 2019) and their subordinates’ work and health outcomes (Skakon et al., 2010). However, the literature has lacked a focus on the experience of middle-level managers who, in their role, are responsible for their subordinates’ experience but, at the same time, are exposed to power and actions from the top management or executive team.

Initial studies have shown how managers become vulnerable to workplace mistreatment. In the literature on bullying, scholars have identified at least four types of bullying based on the characteristics of perpetrators, namely: downward bullying (i.e., bullies are in a higher position than the victim); upward bullying [i.e., subordinates bully a person in a managerial position (Branch et al., 2021)]; horizontal bullying (i.e., bullying happens between peers); and cross-level co-bullying (i.e., colleagues join managers in bullying) (D’Cruz and Rayner, 2013). While much of the extant research has suggested that downward bullying is the most common type of bullying (Einarsen and Skogstad, 1996; Rai and Agarwal, 2017; De Cieri et al., 2019), other studies have raised concerns about the occurrence of upward bullying (Branch et al., 2007; Björklund et al., 2019; Branch et al., 2021). Similar trend was observed in the literature of workplace aggression. In recent years, increasing attention is given to “supervisor-directed aggression” (Tepper et al., 2015; Liang et al., 2022) to studying aggressive experience of managers and suggested that this is resulting from abusive supervision or destructive leadership. Statistics showed that leaders or managers are exposed to mistreatment. For example, Zapf et al. (2011) found that 9.7% of managers experienced mistreatment from their subordinates, whereas Jenkins et al. (2012) found that some managers were accused by their subordinates of bullying for carrying out regular organizational practices which they might not have direct control over or implementing appropriate managerial procedures which in turn caused the managers difficulties in their workplaces, including forced resignation and personal mental health issues.

Much existing evidence has suggested that perpetrators are usually those in a higher position or with authority over employees, that is, managers or senior employees. The formal authority of the manager’s position to oversee rules, maintain order and monitor subordinates might be misused in his/her inappropriate behaviors and actions. However, it is worth noting that the power imbalance might not be restricted to the individual’s position in the formal hierarchy or to his/her functional position. Scholars have argued that sometimes individuals at lower levels could obtain enough informal power to bully their managers or superiors through certain tactics (Branch et al., 2013). For example, spreading rumors or gossip about a manager creates tension with, and a stressor to, that manager. Upward mistreatment could happen in various ways, such as withholding important information from managers, missing deadlines and “slacking off” at work. These ways may differ from what is usually termed “bullying or aggressive behaviour” but indeed is a form of negative act.

Previous studies have constantly shown that employees who experience workplace bullying and/or aggression are more likely to experience emotional problems, take sick leave, be less committed and consider leaving the organization (Einarsen et al., 2018; Boudrias et al., 2021). However, little focus has been directed to the consequences of upward bullying. Nonetheless, as with all other mistreatment experiences, upward bullying and mistreatment are expected to be detrimental to an individual’s health and well-being. They are likely to lead to negative consequences affecting the individual’s daily life, interactions and working experience. For example, in a diary study, Adiyaman and Meier (2022) found that incivility in the workplace was related to feelings of anger and depression. Hence, we expect that:

*H1*: (a) Upward bullying; and (b) aggression are negatively related to middle managers’ positive affect.

*H2*: (a) Upward bullying; and (b) aggression are positively related to middle managers’ negative affect.

### 1.2 Importance of the relationship between PSC and mistreatment

The literature on workplace mistreatment has consistently shown the importance of having a workplace with effective policies for the prevention of aggression and negative actions (Hershcovis et al., 2007, 2020; Han et al., 2022; Ervasti et al., 2023). A workplace with effective conflict management policies and procedures that resolves conflict and tension between the parties involved, will be able to prevent employees from reacting in a negative way (Tuckey et al., 2022), thus ensuring individuals’ fundamental rights and respect for each other (Liefooghe and Mackenzie Davey, 2001). Examples of effective management practices include, leading teams with respect and upholding equity among employees, having regular team meetings to discuss difficult work situations and providing support for worker to solve problems. In line with the work environment hypothesis of mistreatment (Hauge et al., 2007), workplaces with low levels of psychosocial risks, such as work overload, time pressure, negative social relationships and low job control, are also less likely to produce bullying and negative acts. Similarly, literature proposed that organizational norms and intolerance of aggression were likely to reduce aggression at workplaces (Lyubykh et al., 2022). The importance of the work environment versus individual factors is further supported by a meta-analysis which revealed that the correlations between environmental factors and workplace mistreatment are much higher than the correlations of individuals’ characteristics with workplace mistreatment (Han et al., 2022). For example, an incivility climate has a correlation of 0.57 with workplace incivility in comparison to the relationship of neuroticism with incivility which has a correlation of 0.16 (Han et al., 2022). We argue that an incivility climate could be removed by building a psychosocial safety climate (PSC).

Psychosocial safety climate (PSC) refers to the organizational climate that emerges from organizational policies, practices and procedures that focus on the protection of employees’ psychological well-being (Dollard and Bakker, 2010). Due to its nature and focus on employee’s well-being, PSC is one of the most studied climate measures in relation to occupational health and safety (Loh et al., 2019; Yang et al., 2023). Four main principles comprise the PSC concept: (a) management priority given to employees’ psychological health over productivity; (b) management support and commitment given to improving and protecting employees’ psychological health; (c) organizational communication about psychological health-related matters; and (d) organizational participation by, and involvement of, all levels of stakeholders in relation to employees’ psychological health and safety matters (Dollard and Bakker, 2010). These principles suggest that the level of PSC is largely determined by organizational top management and decision makers. Through focusing on the four PSC principles (i.e., each of these domains), a work context could be built that cultivates positive interactions between employees (Kwan et al., 2016), enables effective resources utilization (Loh et al., 2018) and fulfils individual employees’ fundamental needs (Huyghebaert et al., 2018).

The relationship between PSC and upward bullying can be viewed from the perspective of the frustration–anger hypothesis (Escartín et al., 2021). In the literature on workplace mistreatment, scholars have termed these negative behaviors and actions as “negative acts,” “deviant acts” and “counterproductive acts.” These terms suggest a form of behavior resulting from frustration and anger. Displaying and engaging in workplace mistreatment is likely to be a strategy to reveal an individual’s feelings of dissatisfaction toward the work context (Baillien et al., 2011) and their leaders (Wallace, 2009). This is particularly true when the employees lack power or job control to influence or change the stressful or high-risk work environment (Tuckey et al., 2022). Tuckey and colleagues uncovered that ineffective workplace policies and the management system shape a high-risk environment in which workplace mistreatment arises. Scholars have studied the “retaliation” of employees against abusive supervision suggesting that when employees working with disruptive leaders, who engage in sabotaging their subordinates or react negatively toward employees’ reasonable requests, the likelihood of acting poorly toward their leaders increases (Lyubykh et al., 2022). In addition, Lyubykh et al. (2022) further found that workplace intolerance of mistreatment moderated the relationship between abusive supervision and retaliation, such as being rude to their supervisor, yelling, providing false information, or even physical aggressive behaviors including trying to hit their supervisor. Using the frustration–anger perspective, individuals who are frustrated with their experience will react with anger and are more likely to engage in overt aggressive behaviors. That said, if employees are dissatisfied with their work context or leaders, it is possible that they will react with aggressive and bullying behaviors toward their leaders, either in a covert or overt way. Conversely, if employees are working in a favorable work context, aggressive/bullying behaviors are less likely to occur. Hence, high levels of PSC emanating from top management, influential in establishing positive work conditions at lower functional levels, could be associated with lower levels of upward mistreatment.

Upward mistreatment is also a form of unsafe behaviors. In the literature, scholars have widely studied safety behaviors which aim to ensure the safety and health of employees in their workplace. Safety behavior, including safety compliance and safety participation, refers to actions and daily activities that aim to avoid danger, risks, and hazards (Neal and Griffin, 2006). Extending this concept to psychosocial aspects, psychosocial safety behaviors are behaviors that relate to employees’ psychological safety and health, such as being respectful and actively involved in promoting better psychological well-being at work (Bronkhorst, 2015). Safety behaviors research has suggested that employees are likely to engage in safety behaviors and to comply with safety norms when safety climate is at a high level (Jiang et al., 2019). Stated differently, unsafe behaviors are the consequences of an unsafe work environment (climate). Therefore, upward bullying could result from a less psychologically safe work environment, largely due to the existing social context which indicates tolerance for negative actions and less emphasis on respect and care toward others. Hence, PSC could reduce the likelihood of upward bullying through anti-bullying policies (Dollard et al., 2017), effective people management strategies (Tuckey et al., 2022) and encouraging employee voice in relation to undesirable treatment (Kwan et al., 2016).

Therefore, our study examines the influence of PSC as reported by themselves and their subordinates on managers’ experience of upward mistreatment and their well-being. Firstly, we examine the odd ratios of experiencing upward mistreatment when there is low PSC. Secondly, we study the association between managers’ self-perceived PSC, upward mistreatment and their well-being. Thirdly, we explore the link between employees’ perceived PSC and managers’ experience of upward mistreatment.

When PSC is low, particularly when the score is below 37 (score of 60 indicates perfect PSC), research has shown that employees are more likely to develop job strain and depression (Bailey et al., 2015; Dormann et al., 2018; Zadow et al., 2021). A low PSC environment suggests that employees’ well-being is compromised in terms of productivity and performance, hence the environment created is less humane and caring. Therefore it is more likely that mistreatment will occur. As a result, we expect that:

*H3*: The odd ratios of (a) upward bullying; and (b) aggression is higher when PSC is low compared to when PSC is high, as reported by managers and the employees from their organizations.

As a climate construct, PSC is often studied in two different ways, first as a psychological climate and second as an organizational climate. Psychological PSC refers to the individual’s perceptions of their experience of the work context, leaders, and their personal interpretation of the social context in related to the protection of employees’ psychological health and well-being. Organizational PSC is often measured through shared perceptions of employees on their common experience and work environment using the referent-shift consensus model (i.e., asking about a higher-order organisational structure) (Chan, 1998). The convergence process of employees’ perception gives rise to a collective perception of PSC which could be measured at the group or organizational level of analysis. Studies in the literature have explored both psychological and organizational PSC with consistent findings on PSC’s impact as a leading indicator of work stress, job conditions and social relationships (Loh et al., 2020). High levels of PSC reflect a resourceful environment and favorable job conditions that would lead to fewer negative interactions (Tuckey et al., 2022), hence reducing upward bullying and its negative effect on well-being:

*H4*: Middle managers’ perceived psychological and organizational PSC are negatively related to employees’ (a) upward bullying; and (b) upward aggression.

*H5*: Employees’ (a) upward bullying; and (b) upward aggression mediate the relationship of middle managers’ perceived psychological and organizational PSC with managers’ well-being.

In line with our argument that upward mistreatment is an act of retaliation, it is necessary and meaningful to take account of employees’ reported PSC in our hypotheses. As previously mentioned, PSC is an organizational climate which emerges as a collective construct when a certain level of agreement is achieved between organization members. The emergence process of forming an organizational construct is complex; however, the emergent status of a collective construct can be measured by aggregating individual organization members’ reported scores (Chan, 1998). In line with the argument that an emergent construct is a consensus between the organization’s team members, PSC can be measured by assessing the perceptions of other members within the organization. For example, using a split-sample method, Loh et al. (2018) found that PSC boosts the moderating effect of rewards to mitigate the negative impact of emotional demands on employees’ health problems. Similarly, Escartín et al. (2021) found that PSC relates to workplace bullying and emotional exhaustion by linking three different sources of PSC from within an aggregated sample from the same organizations to examine between-group effects. Previous studies established the homology and isomorphism of PSC, suggesting that PSC is a construct that holds similar meanings and functional impact at both psychological (individual) and organizational (aggregated) levels. Using a split-sample method to assess a multilevel construct could improve the validity of the results and reduce single-source bias.

More importantly, research has detected the upward mistreatment phenomenon but have not yet discovered the reason for its emergence. Aligning with the literature of employee retaliation to disruptive supervision and leadership (Liang et al., 2022), we conceptualize upward mistreatment as a way employees act out on their negative experience and anger. This is largely due to the lack of effective policies in handling employees’ stress, frustration, and dissatisfaction, which could be stem from a low PSC environment. Previous studies showed that low PSC is associated with negative emotions such as anger (Idris and Dollard, 2011) and distress. PSC is also an antecedent for effective anti-bullying policies and conflict management system (Dollard et al., 2017). Therefore, by examining employees’ reported PSC, we hypothesize that:

*H6*: Employee-perceived organizational PSC is negatively related to the upward bullying and upward aggression experienced by middle managers.

*H7*: Employees’ (a) upward bullying; and (b) upward aggression mediate the relationship between employees’ perceived organizational PSC and middle managers’ well-being.

## 2 Methods

### 2.1 Respondents and research design

The data were collected from 191 Australian public organizations with 34,953 employees and 7,013 middle managers by an Australian state government agency. The survey is an annual cohort study which examines the status of occupational health and safety in the public sector of an Australian state. Respondents were asked to answer a survey questionnaire on the status of their working conditions, health, motivation, and experience at work. In our study, we focused on responses about PSC, bullying, positive and negative affect. De-identified data were provided to the authors by the agency based on a research agreement. Due to the nature of the anonymity of archival data, the Human Research Ethics Committee of the authors’ university exempted this study from the need for ethics approval. Respondents were employees involved in managing and delivering services across education and training, health care, public administration, public safety, transport and transport infrastructure, utilities management, and arts and culture.

In this study, the sample was first split into managers and employees. Employees’ responses were aggregated to the organizational level. Any organizations with less than five employees were omitted. To ensure optimal power of a multilevel model, we also omitted organizations with less than 10 responses from managers. This resulted in a final sample of 123 organizations with 6,658 managers.

Of the initial 7,013 managers, 34.5% were male; most were aged from 45–54 years; 84% worked full-time while 13.5% were on a fixed-term contract or casual (0.6%). In total, 32.4% of these managers had worked more than 10 years in their current organization. Among the employees, 66.2% were female, mostly aged between 35–44 years old, 29.5% of them had worked more than 10 years in the organization.

### 2.2 Measurements

Psychosocial safety climate (PSC) was assessed using the PSC-4 scale (Dollard, 2019), a shortened version of the PSC-12 scale (Hall et al., 2010) with good psychometric properties. An example of an item is “Senior management show support for stress prevention through involvement and commitment.” A 5-point Likert scale was used (1 = *strongly disagree*; 5 = *strongly agree*). Cronbach’s alpha was α = 0.90 (for managers) and α = 0.91 (for employees).

To assess the experience of *mistreatment*, definitions of “bullying” and “aggression” were given as detailed below:

*Bullying* – Repeated unreasonable behavior directed at an employee that creates a risk to his/her health and safety.

*Aggression* – When a person is abused, threatened, or assaulted in a situation related to his/her work.

Upward bullying and aggression were measured in three separate questions by asking respondents whether they had experienced these behaviors from their subordinates or followers, rating their answer on *Yes* = 1 or *No* = 0.

Positive affect was assessed with two items asking respondents: “How often has your work made you feel (a) enthusiastic and (b) happy?” A 5-point Likert scale was used to measure this response (1 = *rarely*; 5 = *always*). As suggested by Eisinga et al. (2013) that the Spearman-Brown’s coefficient is a better performing reliability index for two-item scale, the coefficient of these two items was *r_sb_* = 0.90.

Negative affect was assessed by asking respondents: “How often has your work made you feel (a) worried and (b) miserable?” with ratings on a 5-point Likert scale (1 = *rarely*; 5 = *always*). Spearman-Brown’s coefficient of these two-items was *r_sb_* = 0.75.

### 2.3 Analysis procedure

The relationships between PSC, upward mistreatment and well-being at individual level were tested using regression in IBM’s SPSS Statistics (SPSS) software and multilevel model were conducted using the M*plus* statistical package. The relationships between upward mistreatment and positive and negative affect were tested in a linear regression model.

To assess the probability of occurrence of upward mistreatment we calculated the odd ratios. The odds ratios of managers reported PSC on upward bullying, aggression and sexual harassment were calculated using logistic regression. The multilevel odds ratio was calculated using multilevel logistic regression by regressing upward bullying and aggression on employees’ reported PSC.

To justify the use of multilevel modeling, we ran several tests including one-way analysis of variance (ANOVA) and intraclass correlation (ICC [1] and ICC[2]) for PSC reported by managers and employees. For PSC as reported by managers, *F* = 3.32, *p* < 0.001, ICC (1) = 0.06, and ICC (2) = 0.71. For employees’ PSC, *F* = 12.48, p < 0.001, ICC (1) = 0.06, and ICC (2) = 0.92. The results supported the collective properties of PSC.

**Figure 1:**
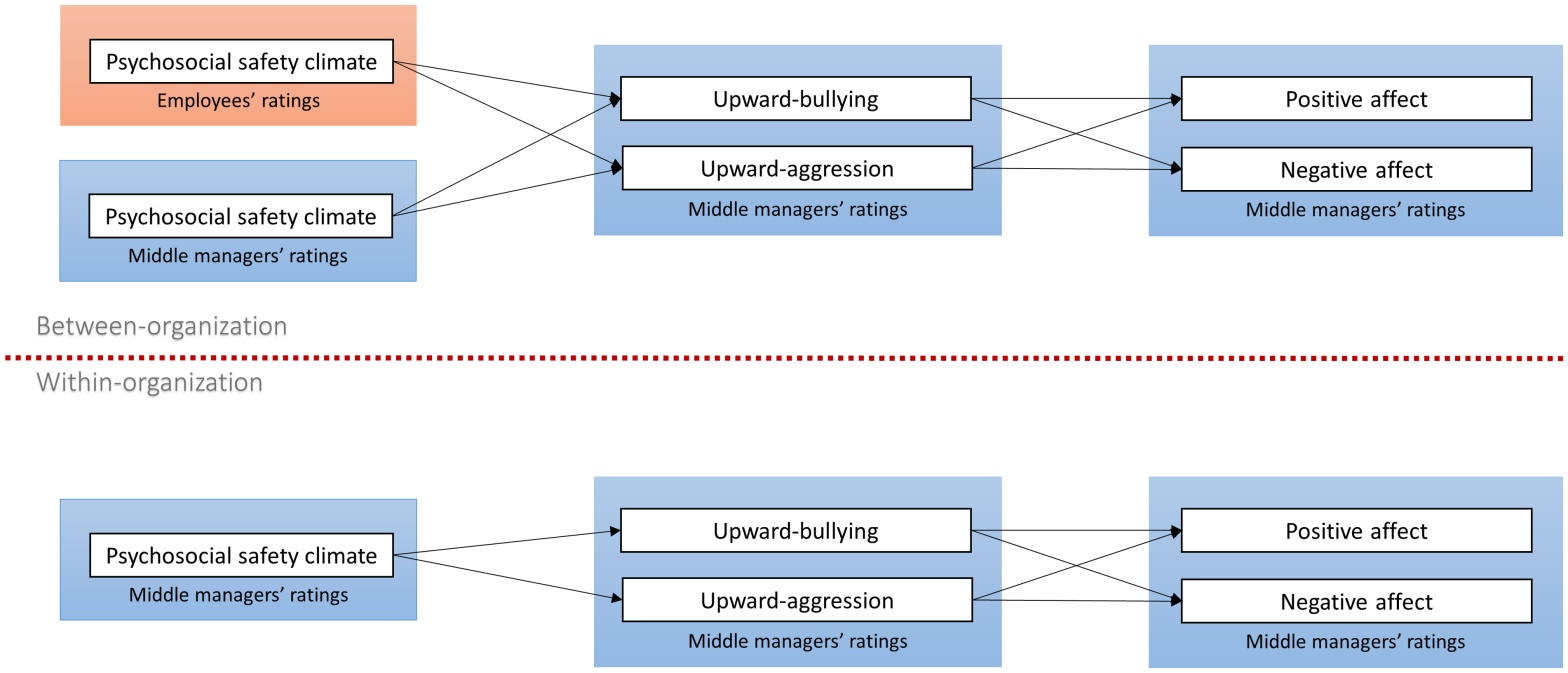
Study model.

We assessed the between-group effects of PSC, as reported by employees and managers, on managers’ experience of upward aggression and bullying using negative binomial multilevel-modeling to account for skewness and excessive variance in the count data. A robust maximum likelihood (MLR) estimator was used with a group-mean centering for Level-1 independent variables. As shown in Figure 1, a multilevel homologous model was tested. Managers’ positive and negative affect were regressed on upward aggression and upward bullying at both between- and within-organizational level. At between-organizational level, upward aggression and bullying were regressed on organizational-level PSC, as reported by employees and managers. At the within-organizational level, upward aggression and bullying were regressed on managers’ self-reported PSC only. We also controlled for managers’ gender, age, and organizational tenure at the within-organizational level. Indirect effects of between- and within-level PSC, upward mistreatment and affects were estimated by bootstrapping. Due to the model’s convergence problems, we ran two separate multilevel models, one with employees’ reported PSC and the other with managers’ reported PSC at the organizational level: all variables and pathways were the same as those at the individual level.

### 2.4 Exploratory analysis

Another common type of mistreatment is sexual harassment, which is different from other harassment such as racial harassment or general gender harassment. Unlike bullying or aggression, sexual harassment involves undesired and offensive sexual-related behaviors, such as sexual jokes and improper invitations that may not need to occur more than once or persistently to be named so (Fitzgerald et al., 1997; Cortina and Areguin, 2021). While the prevalence of sexual harassment is high, particularly among women, sexual harassment directed to managers from subordinates or individuals at lower position could be rare, given that many legal regulations have been established in dealing with sexual harassment. This would then reduce the subtle form of sexual harassment (Hershcovis and Barling, 2010). Indeed, only 0.4% of managers in the current study having reported this. Yet this is a very important issue and should not be neglected, we included it as an exploratory analysis. Similar to bullying and aggression literature, scholars of sexual harassment proposed that situational factors, including social norm hugely influence the likelihood of sexual harassment occurrence (Pryor et al., 1993; Williams et al., 1999), particularly when the organization ignored the problem, tolerates such behaviors, and discourages complaints. Theoretically, PSC is relevant in reducing the likelihood of sexual harassment.

The definition of sexual harassment provided in the survey was as follows.

*Sexual harassment* – Defined as non-consensual or unwelcome sexual behavior that could be expected to make a person feel offended, humiliated, or intimidated. Sexual harassment may be physical, spoken or written and can be directed at, and perpetrated by, persons of any sex or gender. A single incident can constitute sexual harassment, as can a broader pattern of behavior.

## 3 Results

The findings showed some support for our study’s hypotheses. Moreover, the results obtained using multilevel models provided additional support for the relationship between PSC and upward mistreatment. The results are described below.

Hypothesis 1 proposes that experiencing upward mistreatment will negatively relate to positive well-being in managers. Individual-level regression results supported this assumption for upward bullying and upward aggression (Table 1). Upward bullying was negatively related to positive affect (*B* = −0.26, standard error [SE] = 0.05, *p* < 0.001), with similar findings found for upward aggression (*B* = −0.25, SE = 0.04, *p* < 0.001). However, none of these relationships were found to be significant at the organizational level (Table 2). The results supported H1a and H1b at the individual level. Hypothesis 1 was not supported at the organizational level.

**Table 1:** Regression results of upward bullying, aggression, and sexual harassment on managers’ well-being.

Outcomes	Positive affect				Negative affect			
Parameter	*B*	SE	95% CI, [LL;UL]	*p*	*B*	SE	95% CI, [LL;UL]	*p*
Upward bullying → affect	**−0.26**	0.05	[−0.35; −0.17]	< 0.001	**0.59**	0.05	[0.50; 0.69]	<. 001
Upward aggression → affect	**−0.25**	0.04	[−0.33; −0.17]	< 0.001	**0.57**	0.04	[0.49; 0.66]	<. 001
Upward sexual harassment → affect	−0.10	0.13	[−0.35; 0.15]	0.424	0.19	0.13	[−0.71; 0.45]	0.153

Total *N* = 6,658 managers. CI, confidence interval; LL, lower limit; PSC, psychosocial safety climate; SE, standard error; UL, upper limit. Significant results are in bold.

**Table 2:** Negative binomial multilevel results of PSC, upward mistreatment, and affect.

	Model 1				Model 2			
Outcomes	Positive affect				Negative affect			
Parameter	*γ*	SE	*t*	*p*	*γ*	SE	*t*	*p*
Within-level								
Upward bullying → affect	0.01	0.08	0.11	0.913	−0.04	0.13	−0.29	0.770
Upward aggression → affect	−0.04	0.09	−0.49	0.625	**0.39**	0.13	3.12	0.002
PSC_m_ → affect	**0.50**	0.01	45.72	< 0.001	**−0.46**	0.01	−37.08	< 0.001
PSC_m_ → upward bullying	**−0.25**	0.08	−3.29	0.001	**−0.25**	0.08	−3.29	0.001
PSC_m_ → upward aggression	**−0.26**	0.08	−3.94	< 0.001	**−0.26**	0.08	−3.94	< 0.001
Controls								
Gender	**−0.07**	0.01	−6.07	< 0.001	0.01	0.01	1.05	0.295
Age	0.02	0.01	1.82	0.069	**−0.06**	0.01	−5.42	< 0.001
Organizational tenure	**−0.04**	0.01	−5.71	< 0.001	0.01	0.01	0.72	0.469
Within-level indirect effect								
PSC_m_ → upward bullying → affect	−0.00	0.02	−0.11	0.913	0.01	0.03	0.29	0.771
PSC_m_ → upward aggression → affect	0.01	0.02	0.48	0.634	**−0.10**	0.04	−2.40	0.017
Between-level								
Upward bullying → affect	−1.32	1.10	−1.2	0.230	0.19	1.28	0.15	0.881
Upward aggression → affect	1.27	0.95	1.33	0.182	−0.30	1.10	−0.28	0.784
PSC_e_ → affect	−0.04	0.07	−0.55	0.583	−0.02	0.07	−0.26	0.793
PSC_e_ → upward bullying	**−0.71**	0.32	−2.25	0.025	**−0.71**	0.32	−2.25	0.025
PSC_e_ → upward aggression	**−0.61**	0.31	−1.99	0.046	**−0.61**	0.31	−2.00	0.046
Between-level indirect effect								
PSC_e_ → upward bullying → affect	0.94	0.87	1.07	0.283	−0.14	0.91	−0.15	0.880
PSC_e_ → upward aggression → affect	−0.77	0.72	−1.08	0.279	0.18	0.66	0.28	0.781
Model fit								
−2 Log Likelihood	18318.26				19823.73			
AIC	18362.27				19867.41			
BIC	18511.95				20017.41			

Total *N* = 6,658 managers, 123 organizations. AIC, Akaike information criterion; BIC, Bayesian information criterion; PSC_m_, psychosocial safety climate reported by managers; PSC_e_, psychosocial safety climate reported by employees; SE, standard error. Significant results are bold.

Hypothesis 2 proposes that upward mistreatment is positively related to negative affect. Results showed that upward bullying and upward aggression were positively related to managers’ negative affect (upward bullying *B* = 0.59, SE = 0.05, *p* < 0.001; upward aggression *B* = 0.57, SE = 0.04, *p* < 0.001) (Table 1). As with positive affect, upward bullying was not found to be related to managers’ negative affect at the organizational level (Table 2). However, upward aggression was positively related to managers’ negative affect (*B* = 0.39, SE = 0.13, *p* = 0.002). H2a and H2bwere supported at the individual level, with only H2b supported at the organizational level.

We calculated the odds ratio of PSC on upward mistreatment (Tables 3, 4). At the individual level, psychological PSC reduced upward bullying and aggression (*B* = −0.27, SE = 0.07, *p* < 0.001; *B* = −0.28, SE = 0.07, *p* < 0.001, respectively). The odds ratio of managers’ reported PSC on upward bullying was 0.77 while, for upward aggression, it was 0.76 at the individual level, indicating that managers working in a higher PSC organization 23 and 24% less likely to experience upward bullying and upward aggression, respectively. At the organizational level, the odds ratio for employees’ reported organizational PSC on upward bullying was 0.74 (26%) while, for upward aggression, it was 0.78 (22%). To ease interpretation, we divided 1 with the odd ratios and found that as compared to high PSC group, managers in low PSC organization were about 1.28 to 1.35 times more likely to be bullied or treated aggressively by their subordinates at individual and organizational levels. This finding supported H3.

**Table 3:** Results of logistic regression and odds ratios of managers’ individual perceptions of PSC on upward mistreatment.

Outcomes	−2 Log likelihood	*R^2^*	*B*	SE	Wald	*p*	Exp(B) [95% CI, LL;UL]
Bullying	1697.715	0.008	**−0.266**	0.074	12.816	< 0.001	**0.766 [0.663; 0.887]**
Aggression	2054.223	0.010	**−0.279**	0.066	17.951	< 0.001	**0.757 [0.665; 0.861]**
Sexual Harassment	351.984	0.006	−0.284	0.191	2.224	0.136	0.753 [0.518;1.093]

Total *N* = 6,658. SE, standard error; CI, confidence interval; Exp(B), odds ratio; LL, lower limit; UL, upper limit. Significant results are in bold.

**Table 4:** Results of multilevel logistic regression and odds ratios of employees’ perception of PSC on upward mistreatment.

Outcomes	*B*	SE	*t*	*p*	Exp(B) [95% CI, LL;UL]
Bullying	**−0.300**	0.064	−4.706	< 0.001	**0.741 [0.649; 0.834]**
Aggression	**−0.251**	0.062	−4.033	< 0.001	**0.778 [0.683; 0.873]**
Sexual Harassment	−0.271	0.016	−1.69	0.091	0.763 [0.524; 1.002]

Total *N* = 123 organizations. SE, standard error; CI, confidence interval; Exp(B), odds ratio; LL, lower limit; UL, upper limit. Significant results are in bold.

Hypothesis 4 suggests that managers’ PSC is negatively associated with upward bullying and upward aggression at both the within- (individual) and between-organizational level, with H5 proposing a mediation pathway to managers’ well-being (Table 2). At the individual level, results showed that managers’ reported psychological PSC had an indirect effect on negative affect through upward aggression (*B* = −0.10, SE = 0.04, *p* = 0.017). Contrary to our expectation, managers’ perceived organizational PSC was not related to upward bullying and aggression (see Table 5). However, when managers’ reported psychological PSC was included in the model, the relationships between (a) upward bullying and positive affect; (b) upward aggression and positive affect; and (c) upward bullying and negative affect were no longer significant, suggesting a direct effect of PSC rather than a mediated pathway. Similarly, the link of upward bullying on negative affect was not found in the model that included managers’ reported PSC (see Table 2). The results supported H4 but not H5.

**Table 5:** Negative binomial multilevel results of PSC reported by managers and upward mistreatment.

Parameter	Model A			
	*γ*	SE	*t*	*p*
Within-level				
PSC_m_ → upward bullying	**−0.29**	0.08	−3.80	< 0.001
PSC_m_ → upward aggression	**−0.29**	0.06	−4.67	< 0.001
Between-level				
PSC_m_ → upward bullying	0.02	0.31	0.05	0.960
PSC_m_ → upward aggression	0.06	0.27	0.21	0.834
Model fit				
−2 Log Likelihood	3586.82			
AIC	3598.82			
BIC	3639.64			

Total *N* = 6,658 managers, 123 organizations. AIC, Akaike information criterion; BIC, Bayesian information criterion; PSC_m_, psychosocial safety climate reported by managers; SE, standard error. Significant results are bold.

**Figure 2:**
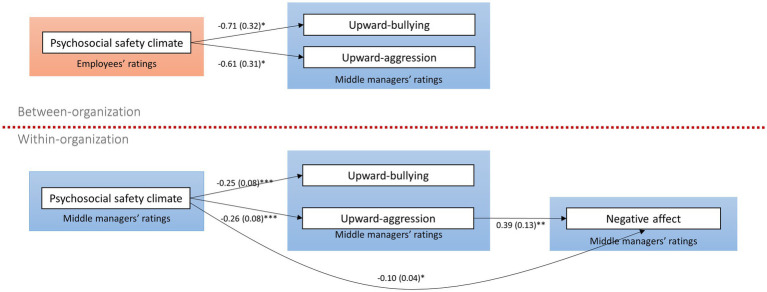
Results model.

At the organizational level, employees’ higher perceived organizational PSC was related to lower upward bullying (Model 1: -0.71, SE = 0.32, *p* = 0.025; Model 2: -0.87, SE = 0.03, *p* < 0.001) and upward aggression (Model 1: -0.61, SE = 0.31, *p* = 0.046; Model 2: -0.83, SE = 0.28, *p* = 0.003). However, no indirect effect was found. The findings supported H6 but not H7. The final model is shown in Figure 2.

In our exploratory analysis for sexual harassment, we could not find any significant relationship between upward sexual harassment to negative and positive affect, nor a relationship between PSC and upward sexual harassment, at both individual- and organizational- level.

## 4 Discussion

The existing literature on workplace mistreatment largely portrays managers as the perpetrators because of substantial evidence of bullying or downward mistreatment by managers yet neglects the possibility of managers being a target themselves. Nonetheless, managers are not immune from being mistreated, particularly by upward mistreatment. The current study examined the antecedents of upward bullying, and aggression by arguing that psychosocial safety climate (PSC) is an important organizational contextual antecedent that could reduce upward bullying and other types of upward mistreatment. Our study examined the unsafe behavior of employees and suggested the importance of cultivating a healthy workplace to reduce negative social interactions and to prevent feelings of frustration and anger among employees. The current study’s findings supported this notion, revealing that employees’ reported PSC, but not managers’ reported PSC, was positively related to upward bullying and aggression. As such the findings suggests that some individual employees will react with aggressive deviant behaviors if their organization fails to address their health and well-being. If a healthy workplace is not created, our findings suggest that this not only affects employees, as confirmed in previous research, but also affects managers owing to retaliation from frustrated subordinates. Splitting the sample and computing the aggregated PSC score from a separate source (i.e., from employees, but not from managers) further supported the notion that PSC is the lead indicator of work conditions and well-being, contributing to managers’ positive and negative affect. Reports by employees and managers indicated that PSC is highly associated with upward mistreatment, particularly in the form of bullying and aggression experienced by managers. Importantly, employees’ organizational PSC revealed a significant impact on managers’ experience of upward mistreatment but not on organizational PSC as reported by managers. The results reflect the perceptions of subordinates on the work environment is a more important risk factor for the prevalence of negative behaviors toward their leaders. Again, this supported our notion that upward mistreatment is a result of unsafe psychosocial behaviors resulting from a low level of psychosocial safety climate (PSC).

Of the eight tested indirect pathways (four within- and four between-organizational level), we found that upward aggression was the only upward mistreatment to mediate the relationship between managers’ reported psychological PSC and managers’ negative affect at the within-organizational level. In other within-organizational pathways, results suggested that PSC reported by managers or employees had a direct effect on managers’ positive or negative affect rather than via a mediator. The results also suggested that PSC is directly related to managers’ well-being above and beyond the impact of bullying and that it accounts for a larger variance in managers’ positive and negative affect. In other words, high perceived PSC is beneficial both for reducing the likelihood of bullying and for providing a potential benefit for managers’ psychological well-being.

### 4.1 Theoretical implications

Our study contributes to the literature on upward bullying and mistreatment. The current study expands the frustration–anger theory of aggression (Escartín et al., 2021) by theorizing that workplace mistreatment is a way to “retaliate” in a negative work context. The findings suggest that employees’ frustration and anger would lead to aggressive behaviors toward their leaders (i.e., their managers) as a way of reacting to the negative work environment. In addition, we propose that upward bullying and mistreatment are unsafe actions resulting from an organization’s unhealthy situation. The current study’s findings also expand the theory on upward bullying, suggesting that upward bullying is an emergence process through which individual employees provide cues and feedback to the organization on their frustration and dissatisfaction. The findings further expand the theory on bullying by including PSC as the antecedent of negative actions, unsafe social interaction and conflict escalation between employees and managers in the absence of an anti-bullying or anti-conflict climate. We extend the literature on PSC emergence, providing evidence that emergent collective PSC is important with a significant impact on other personnel, including managers, in the organization.

### 4.2 Practical implications

The findings of the current study provide some important insights in relation to different types of workplace mistreatment. The current findings found that tackling the issue of managers experiencing upward aggression could help to nurture a workplace that enables managers to fully perform their tasks and that protects them from psychological health issues. However, the findings are slightly different for bullying. While bullying is not at all desirable, the experience of upward bullying by middle-level managers does not seem to be detrimental to their wellbeing. It is possible that resources to manage undesirable situations are easily accessible by them. Specifically, for bullying and aggression from subordinates, employers should be aware that this may reflect a way of employees acting out and voicing their dissatisfaction against negative working environment, such as low PSC. Hence, employers should exert effort to prevent upward mistreatment by establishing a high-level PSC workplace.

The current study also supports that better communication and feedback systems could assist employees to report on poor PSC without acting out through upward mistreatment. Improving PSC could be achieved by practicing the PSC principles of communication, priority setting, participation, involvement, and commitment. This can be done by providing training and workshops for middle managers, introducing and educating them about the concepts of PSC (Dollard and Bailey, 2021), supporting them to enact effective people management practices (such as how to deal with underperforming workers with respect and be responsive to workers’ requests for proper entitlements; Tuckey et al., 2022). Policies in relation to conflict management, such as anti-bullying procedures, effective reporting system and protection for whistle blowers, could nurture a workplace with high protection not only for employees but also for managers. It is also important for top management to support their middle managers in enacting PSC principles and values and to prevent them from experiencing unsafe treatment.

## 5 Limitations and future research

Some limitations in the current study should be acknowledged. The data did not allow us to establish the causal relationship of PSC with upward bullying and aggression. With a one-time data collection, we tried to minimize bias through the linkage of two different sources of responses, thus providing reliable results. In addition, the occupational health psychology literature has reported criticism of the use of self-reported or single-source data. Previous studies often reported significant discrepancies between employees’ and managers’ reports on the work environment and psychosocial risks; however, our study’s findings reported strong coherence on the impact of PSC, as perceived by employees and managers, on the likelihood of upward bullying and managers’ well-being. Future studies should further examine the impact of PSC on upward bullying over time by considering a longitudinal design and time-lagged analysis.

While our study focused on the exposure of upward mistreatment from the manager’s perspective (i.e., the victim) and its link with employees’ perceptions (i.e., the potential perpetrators) of their work environment, it is also necessary to expand the study to understand how the organization manages and reacts when upward mistreatment is reported. In addition, understanding the duration of, and time of exposure to, upward mistreatment will provide further insights on how conflict escalates into bullying and aggression. Although our study establishes the first insights and knowledge on the relationship between PSC and upward mistreatment, scholars have suggested that the frequency and duration of workplace mistreatment could provide more in-depth understanding of its impact and effects (Notelaers and Van der Heijden, 2019). More research is required to determine how the process of conflict escalation unfolds over time (Einarsen et al., 2018; Taris, 2022).

Moreover, to establish a strong causal relationship and to uncover the underlying mechanism of upward mistreatment, scholars should not neglect the influence of individual differences and varied perspectives from victim, perpetrator, or bystander. Our study suggested that a situational factor (i.e., PSC) is highly associated with workplace mistreatment, some studies have discussed individual factors of mistreatment (Coyne et al., 2003; Baughman et al., 2012; Hülsheger et al., 2021), and more importantly how individuals react differently to workplace mistreatment (Matthiesen and Einarsen, 2007). For example, instead of aggressive retaliatory behaviors, mistreatment victims may start to withdraw or reduce their commitment at work, termed as “quite quitting” in recent literature (Mahand and Caldwell, 2023). Future research should consider assessing both situational and personal factors of enacting and reacting to workplace mistreatment simultaneously to gain deeper understanding on how to tackle workplace mistreatment.

In the current study, on an exploratory basis, we did not find any empirical support for the impact of PSC on sexual harassment. While this might be due to low responses in sexual harassment, with 0.4% of managers having reported this, the construct of sexual harassment could be a highly specified phenomenon as compared to bullying and aggression that requires special attention and might be involved with even more complicated organizational social factors (Pryor et al., 1993). In addition, most of the time the perpetrators of sexual harassment were someone from higher ranking than the victims, it is less likely to observe an upward sexual harassment as what we included in the current study. In addition, sexual harassment is also a legal concept that laws and regulations have been implemented to prohibit in many countries, it is possible that this helps reducing the prevalence of sexual harassment at work. However, some scholars have suggested to expand the concept to include gender harassment (Cortina and Areguin, 2021) and urge to think about the issue from a broader “gender-related” perspective (Fitzgerald and Cortina, 2018), and how a gender harassment escalate into sexual harassment. Align with this, organizational structure, such as the proportion between male vs. female, job positions that traditionally dominant by men (e.g., engineers, construction workers) might have interaction effects with PSC to contribute to gender and sexual harassment.

## 6 Conclusion

Upward bullying and aggression are forms of mistreatment experienced by managers; yet they can potentially be prevented by establishing a desirable work environment with PSC at a high level. The current study’s results were confirmed using both managers’ self-perceived psychological PSC and perceived organizational PSC of employees. The current study’s findings support the need to establish a psychologically healthy workplace to reduce the potential for workplace mistreatment and to protect managers from employees’ negative actions. It is evident that a healthy and favorable work context is important to ensure managers’ well-being and to prevent aggressive reactions from employees potentially arising from their frustration and dissatisfaction. Employers and organizational management teams should focus their attention on the organizational context and safety management system in their efforts to protect managers and employees from psychological threats and workplace mistreatment. Future studies on upward mistreatment should expand to include PSC as a preventive measure of middle managers’ experience of unsafe psychosocial acts from their subordinates.

## Data availability statement

The data analyzed in this study is subject to the following licenses/restrictions: the dataset is proprietary and confidential. Requests to access these datasets should be directed to MD, maureen.dollard@unisa.edu.au.

## Ethics statement

The studies involving humans were approved by University of South Australia Human Research Ethics Committee. The studies were conducted in accordance with the local legislation and institutional requirements. The ethics committee/institutional review board waived the requirement of written informed consent for participation from the participants or the participants’ legal guardians/next of kin because the data has been achieved and no personal information was included.

## Author contributions

ML: Conceptualization, Data curation, Formal analysis, Methodology, Writing – original draft. MD: Conceptualization, Funding acquisition, Methodology, Resources, Supervision, Writing – review & editing.
